# Content Adaptive Lagrange Multiplier Selection for Rate-Distortion Optimization in 3-D Wavelet-Based Scalable Video Coding

**DOI:** 10.3390/e20030181

**Published:** 2018-03-08

**Authors:** Ying Chen, Guizhong Liu

**Affiliations:** School of Electronic and Information Engineering, Xi’an Jiaotong University, Xi’an 710049, China

**Keywords:** rate-distortion optimization, mode decision, scalable video coding, Lagrange multiplier, 3-D wavelet-based SVC, motion-compensated temporal filtering

## Abstract

Rate-distortion optimization (RDO) plays an essential role in substantially enhancing the coding efficiency. Currently, rate-distortion optimized mode decision is widely used in scalable video coding (SVC). Among all the possible coding modes, it aims to select the one which has the best trade-off between bitrate and compression distortion. Specifically, this tradeoff is tuned through the choice of the Lagrange multiplier. Despite the prevalence of conventional method for Lagrange multiplier selection in hybrid video coding, the underlying formulation is not applicable to 3-D wavelet-based SVC where the explicit values of the quantization step are not available, with on consideration of the content features of input signal. In this paper, an efficient content adaptive Lagrange multiplier selection algorithm is proposed in the context of RDO for 3-D wavelet-based SVC targeting quality scalability. Our contributions are two-fold. First, we introduce a novel weighting method, which takes account of the mutual information, gradient per pixel, and texture homogeneity to measure the temporal subband characteristics after applying the motion-compensated temporal filtering (MCTF) technique. Second, based on the proposed subband weighting factor model, we derive the optimal Lagrange multiplier. Experimental results demonstrate that the proposed algorithm enables more satisfactory video quality with negligible additional computational complexity.

## 1. Introduction

With the rapid development of video services in recent years, how to efficiently compress video sequences has been considered as a very challenging task for transmitting video data over heterogeneous networks. To meet this demand, scalable video coding (SVC) has become a simple and flexible solution to enable seamless delivery by offering three different kinds of scalabilities, namely, temporal scalability, spatial scalability, and quality scalability [1,2,3,4]. Generally speaking, there are two main categories on SVC: discrete cosine transform (DCT)-based hybrid video coding SVC and wavelet-based SVC.

Owing to the intrinsic localization and multiresolution features of the discrete wavelet transform (DWT), video codecs based on motion-compensated three-dimensional (3-D) DWT have been studied extensively for use in SVC [5,6,7,8]. 3-D wavelet-based SVC provides a natural way in producing embedded bitstreams with full scalability and fine granularity for in-network adaptation [9,10]. In the 3-D wavelet-based SVC, temporal redundancy across frames is exploited by adopting the motion-compensated temporal filtering (MCTF) framework [11,12,13,14], and spatial redundancy inside a frame is utilized by 2-D spatial transform. Such codecs do not suffer from the drift problem often exhibited by the DCT-based SVC codecs with their incorporated feedback loops. Consequently, over the last few years, much research has been devoted to the 3-D wavelet video coding, and it is meaningful to further explore its properties for developing a best coding approach for 3-D wavelet-based SVC.

As in hybrid video coding-based SVC, rate-distortion optimization (RDO) plays a significant role in SVC in choosing the most suitable coding parameters under communication bandwidth constraints. A critical problem residing in RDO is the selection of the Lagrange multiplier that controls the RD trade-off [15,16,17,18,19,20,21,22,23]. Sullivan and Wiegand first derived the relationship among the Lagrange multiplier, distortion and bitrate. This simple and effective formula has been extensively adopted in hybrid video coding [24,25]. Although the conventional Lagrange multiplier selection techniques have been developed and widely adopted in hybrid video coding, the existing formulations are not applicable to 3-D wavelet-based SVC. This is principally due to the fact that the Lagrange multiplier is determined by the quantization step. Different from hybrid video coding-based SVC where the quantization steps are implied in the base layer, quantization for wavelet-based SVC is usually approached in an embedded manner rather than using explicit quantization steps. Accordingly, in absence of the quantization step, the conventional Lagrange multiplier cannot be directly applied to 3-D wavelet-based SVC.

The Lagrange multiplier selection technique used to solve the RDO problem is an important feature that contributes to the success of current video coding. However, solving the optimal Lagrange optimization problem in 3-D wavelet coding is more complicated because the energy difference between the pixel and wavelet domains is not conserved after applying the bi-orthogonal wavelet transform and MCTF technique. The latter leads to the intrinsic subband coupling across various temporal subbands during motion aligned temporal filtering. Moreover, the open-loop prediction structure employed in 3-D wavelet-based SVC further complicates the issue of Lagrange multiplier selection. This issue has been solved by assigning different weighting factors to the subbands, resulting in degrading unpleasant quality fluctuations [18,19,26,27,28]. For the Haar filter used in temporal filtering structure, Ohm proposed a method to derive various quantization weights associated with low-frequency and high-frequency subbands [29]. Xiong et al. have obtained weighting factors for other longer temporal filters, such as 5/3 and 9/7 filters [30,31]. Peng et al. derived the weighting factors from the subscriber’s preference for different resolutions [32,33]. In theory, the subband distortions presented in the reconstructed frame through inverse wavelet transform can be accurately derived from the filter-based weighting factors. However, all the temporal subbands in each temporal decomposition level have diverse features from each other. As a consequence, it is necessary to consider both the diverse content characteristics of the temporal subbands and the impact of subband coupling in the Lagrange multiplier selection process.

Inspired by the methods mentioned above, we propose a content adaptive Lagrange multiplier selection algorithm for RDO in 3-D wavelet-based SVC. During the RDO process, the wavelet filter types, subband coupling phenomenon, and temporal subband content information are all taken into account to adaptively compute the Lagrange multiplier. Our strategy aims to not only accurately select the Lagrange multiplier of each temporal subband in the MCTF decomposition level, but also yield better video quality. By performing the proposed algorithm, our codec clearly outperforms the existing well-known 3-D wavelet-based SVC coding schemes with higher PSNR gains and much lower video quality variations through the whole reconstructed video sequence.

The rest of this paper is organized as follows: Section 2 presents a brief overview of related work. The proposed content-adaptive Lagrange multiplier selection is introduced in detail in Section 3 with its performance evaluation provided in Section 4. Conclusions and discussions are drawn in Section 5.

## 2. Related Work

In this section, we review relevant background information related to the Lagrange multiplier selection in the wavelet-based SVC. The reasons for the failure of the conventional Lagrange multiplier selection in 3-D wavelet-based SVC are also addressed.

### 2.1. System Model

The representative motion compensated embedded zero block coding (MC-EZBC) scalable video coder [34] is a highly efficient member of the existing 3-D wavelet-based SVC schemes. However, the coding efficiency of MC-EZBC in the range of low bitrates is far from satisfactory. To improve this deficiency, Wu et al. put forward the well-known enhanced motion-compensated embedded zero block coding (ENH-MC-EZBC), which retains its excellent rate-distortion performance at high bitrates and achieves significant improvement at low bitrates and/or low resolutions [35].

**Figure 1:**
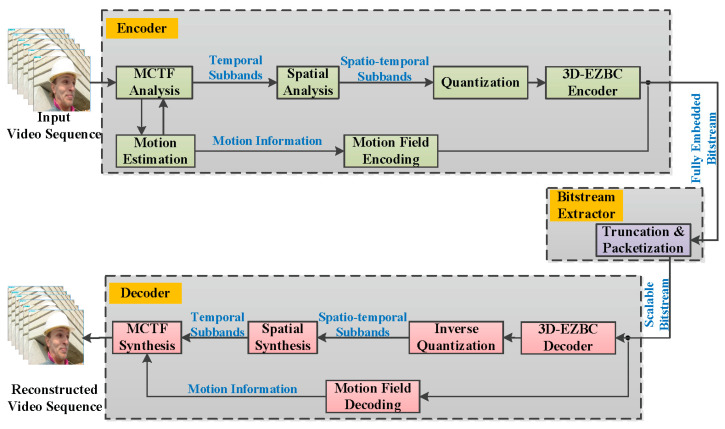
Block diagram of the ENH-MC-EZBC codec system model.

In our work, the proposed algorithm is designed for the popular ENH-MC-EZBC considering quality scalability. Our choice of this codec system is motivated by the fact that ENH-MC-EZBC incorporates all the advanced encoding tools found in the state-of-the-art video coding schemes and obtains the excellent coding performance both at low and high bitrates. The ENH-MC-EZBC codec system model contains three parts: encoder, bitstream extractor, and decoder, which is shown in Figure 1. In the encoder, a motion compensated 3-D subband/wavelet transform naturally partitions the input video sequence into a range of spatiotemporal resolutions. We then use 3D-EZBC to encode the resulting spatio-temporal subbands. All the motion fields are coded as side information by using lossless spatial differential pulse code modulation (DPCM) and adaptive arithmetic coding. In the bitstream extractor, the fully embedded bitstream is truncated based on both user preferences and network conditions to generate a highly flexible scalable bitstream to meet specific applications. At the decoder, the respective reverse operations are carried out to reconstruct video sequences.

### 2.2. Lagrange Optimization and Lagrange Multiplier Selection

The Lagrange optimization technique provides a systematic way to solve the constrained RDO problem, which aims at selecting the optimal coding parameter that minimizes the overall distortion measure subject to a given target bitrate restriction. 

More details on Lagrange optimization technique have been discussed in [16,20,36]. This technique is well known in optimization problems where the cost and objective functions are continuous and differentiable. Everett’s contribution [37] demonstrated that the Lagrange optimization technique could also be used for discrete optimization problems, with no loss of optimality if a solution exists with the required budget; i.e., as long as there exists a point in the convex hull that meets the required budget.

Let {Para} denote the coding parameter set, including motion estimation, mode decision, MCTF decomposition levels, etc. For mathematical convenience, the rate control problem can be formulated as: Minimize the distortion D, subject to a target rate $R_{T}$:

(1) $${\left\{\mathrm{Para}\right\}}_{opt}=\arg\;\underset{\left\{\mathrm{Para}\right\}}{\min}\;D=\arg\;\underset{\left\{\mathrm{Para}\right\}}{\min}\;\sum_{i=1}^{K}d_{i}\;s.t.\;\sum_{i=1}^{K}r_{i}\leq R_{T},$$

where $d_{i}$ and $r_{i}$ are the distortion and bitrate for the ith (i=1,2,⋯K) coding unit, respectively; K is the total number of coding units involved and $R_{T}$ the available bitrate constraint. 

In view of the Lagrange optimization, the above constrained optimization problem (1) can be converted into an unconstrained form as:

(2) $${\left\{Para\right\}}_{opt}=\arg\;\underset{\left\{\mathrm{Para}\right\}}{\min}\;J=\arg\;\underset{\left\{\mathrm{Para}\right\}}{\min}\;(D+\lambda R)=\arg\;\underset{\left\{Para\right\}}{\min}\;\sum_{i=1}^{K}\left(d_{i}+\lambda r_{i}\right),$$

where J=D+λR is the Lagrange cost function, and λ is the so-called Lagrange multiplier that weights the relative importance between $d_{i}$ and $r_{i}$. The optimal coding parameter set for all coding units can be determined by minimizing the Lagrange cost function as expressed in Equation (2). Consequently, how to determine λ becomes a key problem in Lagrange optimization. To have a better solution to the unconstrained problem, much effort has been placed on the research of the Lagrange multiplier selection. One may attain the λ using bisection search [38,39]. In RDO for video coding, however, a more computationally efficient approach is usually favorable to determine the Lagrange multiplier.

Rather than empirically solving the problem of Lagrange multiplier selection as in [38,39], the λ in video coding can be determined using an R-D function. Due to the convexity of the R-D curve, the optimal slope λ matched to the desired R can be easily obtained using standard convex search techniques [24,40]. For each coding unit, the point on the R-D curve that minimizes the Lagrange cost is that point at which the line of absolute slope λ is tangent to the convex hull of the R-D curve.

Note that the R-D curve is convex and non-increasing in video coding, and if we assume that both R and D are differentiable everywhere, the minimum of the Lagrange cost function J is given by setting its derivative to zero, i.e.:

(3) $$\frac{\partial J}{\partial R}=\frac{\partial D}{\partial R}+\lambda =0,$$

which yields:

(4) $$\lambda =-{\frac{\partial D}{\partial R}|}_{R=R_{T}}.$$

where $R_{T}$ is the target bitrate. A given value of λ yields an optimal solution ${\left\{Para\right\}}_{opt}\left(\lambda \right)$ to the original RDO problem (1) for a particular value of $R\left({\left\{Para\right\}}_{opt}\right)$.

### 2.3. Problem Formulation

**Figure 2:**
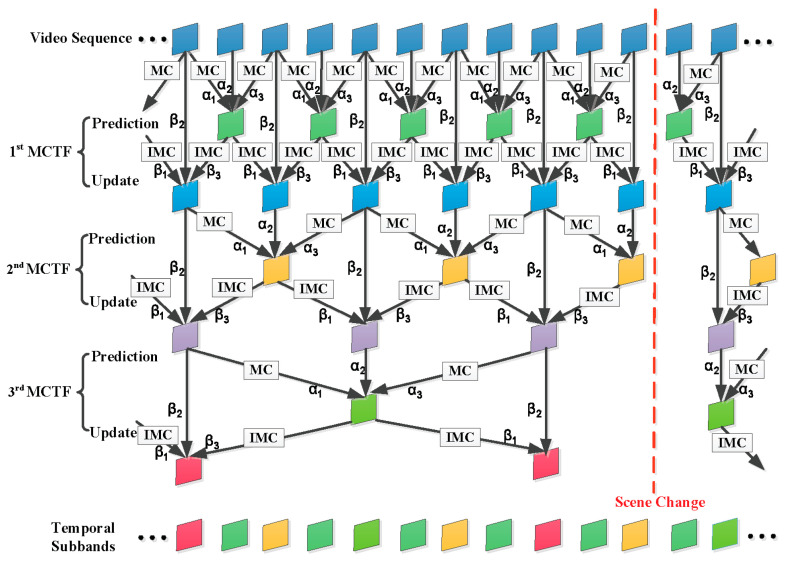
Lifting-based MCTF framework with adaptive switching based on Haar and 5/3 filters.

In 3-D wavelet-based SVC, the temporal decomposition is efficiently refined by adopting the MCTF technique. The ENH-MC-EZBC scalable video coder utilizes an adaptive lifting-based MCTF framework with switching between LeGall and Tabatabai (LGT) 5/3 and Haar 2/2 filters to exploit the temporal redundancies between successive frames [41]. During the MCTF process, video frames are filtered into low-frequency (L) and high-frequency (H) subbands. The process to generate the temporal high- and low- subbands is called the “prediction” and “update” step, respectively. Figure 2 illustrates the prediction and updates steps for the analysis stage of the three-level adaptive MCTF decomposition. MC and IMC are motion compensation and inverse operators, respectively. Scene change information is reflected in the choice of the filter bank, which propagates to the lower temporal levels. The coefficients $\alpha_{\tau }$ and $\beta_{\tau }$(τ=1,2,3) along the branches are filter-based weighting factors. Since 3-D wavelet-based SVC is usually based on the underlying open-loop MCTF structure, its RD performance is further complicated by the inherent problem of the propagation of the quantization errors along the temporal wavelet decomposition tree [42]. Therefore, the selection of the Lagrange multiplier for wavelet-based SVC should be addressed differently.

## 3. Proposed Lagrange Multiplier Selection Algorithm

In this section, we introduce the proposed content adaptive Lagrange multiplier selection algorithm for the 3-D wavelet-based SVC codec.

### 3.1. Lagrange Multiplier Selection Bottlenecks in 3-D Wavelet-Based SVC

Lagrange multiplier based mode decision is one of the most important technologies in SVC [43]. In the adaptive MCTF framework of 3-D wavelet-based SVC, each temporal subband frame in Figure 2 is assigned to one out of four frame modes, including “bi-direction” (denoted as $m_{1}$), “uni-left” (denoted as $m_{2}$), “uni-right” (denoted as $m_{3}$) and “intra” (denoted as $m_{4}$) modes. The ENH-MC-EZBC SVC codec incorporates an optional rate-distortion (R-D) optimized mode decision algorithm. The goal of an R-D optimized mode-selection algorithm is to choose the best mode from available coding frame modes. The mode that minimizes distortion subject to a rate constraint is chosen as the best frame mode. Let $F=(f_{1},f_{2},f_{3},\ldots ,f_{K})$ denote a group of K frames. For a vector of coding mode allocations $M=(m_{1},m_{2},m_{3},m_{4})$ and a bitrate constraint $R_{T}$, this optimization problem can be expressed by:

(5) $$M^{*}=\arg\;\underset{M}{\min}\;D(F,M),\;s.t.\;R(F,M)\leq R_{T},$$

where $M^{*}$, D(F,M) and R(F,M) denote the vector of best mode allocations, distortion metric, and coded bitrate. This may be written as an unconstrained problem using a Lagrange optimization method as:

(6) $$M^{*}=\sum_{i=1}^{K}\arg\;\underset{M}{\min}\;J(F_{i}),$$

where $J(F_{i})$ is the Lagrange cost for the ith frame and is given as:

(7) $$J(F_{i})=D(F_{i},M_{i})+\lambda \cdot R(F_{i},M_{i}),$$

where $D(F_{i},M_{i})$ is the distortion term between the current frame and its reference frame, and $R(F_{i},M_{i})$ the bitrate term representing the expected number of bits allocated to the ith frame, with a specific λ.

Accurate R-D model is crucial in the determination of the Lagrange multiplier. It is worthwhile to mention that the R-D relationships can be quite different for various temporal subbands (T-bands). Unfortunately, it cannot to maximize coding efficiency through utilizing a fixed Lagrange multiplier at each temporal layer. Consequently, the conventional Lagrange multiplier selection method is not optimal as it does not consider the content characteristics of the T-bands.

### 3.2. Analyzing the Distortion Relationship between Temporal Subbands and Reconstructed Frames

The distortion fluctuation exhibited by the 3-D wavelet-based SVC codecs can be better understood by analyzing the distortion propagation during MCTF. In the ENH-MC-EZBC codec, the adaptive MCTF framework has been implemented using either the Haar or the 5/3 filter to improve the coding performance. As shown in Figure 2, the coefficients $\alpha_{\tau }$ and $\beta_{\tau }$ (τ=1,2,3) along the branches are filter-based weighting factors, which are given in Table 1.

In the Haar MCTF, the original frames are filtered temporally with a two-tap Haar filter along the motion trajectory. For the connected pixels, the low-frequency subbands and high-frequency subbands are implemented by the following lifting structure:

(8) $$H_{i}\left(m,n\right)=I_{2i+1}\left(m,n\right)-{\tilde{I}}_{2i}\left(m-d_{m},n-d_{n}\right),$$

(9) $$L_{i}\left(m-{\bar{d}}_{m},n-{\bar{d}}_{n}\right)=I_{2i}\left(m-{\bar{d}}_{m},n-{\bar{d}}_{n}\right)+\left(1/2\right)\cdot {\tilde{H}}_{i}\left(m-{\bar{d}}_{m}+d_{m},n-{\bar{d}}_{n}+d_{n}\right).$$

For the 5/3-based MCTF, the temporal analysis can be written as:

(10) $$H_{i}\left(m,n\right)=I_{2i+1}\left(m,n\right)-\left(1/2\right)\cdot {\tilde{I}}_{2i}\left(m-d_{m},n-d_{n}\right)-\;\left(1/2\right)\cdot {\tilde{I}}_{2i+2}\left(m+d_{m},n+d_{n}\right),$$

(11) $$L_{i}\left(m-{\bar{d}}_{m},n-{\bar{d}}_{n}\right)=I_{2i}\left(m,n\right)+\left(1/4\right)\cdot {\tilde{H}}_{i}\left(m-{\bar{d}}_{m}-d_{m},n-{\bar{d}}_{n}-d_{n}\right)+\left(1/4\right)\cdot {\tilde{H}}_{i-1}\left(m-{\bar{d}}_{m}+d_{m},n-{\bar{d}}_{n}+d_{n}\right),$$

where $H_{i}$ and $L_{i}$ denote the temporal high- and low-frequency subbands of the ith (i=1,2,⋯K) frame of a video sequence, respectively; $I_{2i}(m,n)$ represents the pixel value at position (m,n) in frame $f_{2i}$; ${\tilde{I}}_{2i}\left(m-d_{m},n-d_{n}\right)$ and ${\tilde{I}}_{2i+2}\left(m+d_{m},n+d_{n}\right)$ denote the interpolated values of pixel (m,n) in the frame 2i and 2i+2, respectively. $\tilde{H}$ is the interpolated value of pixel in the high-frequency frame; $\left(d_{m},d_{n}\right)$ is the motion vector in frame $f_{2i}$; ${\bar{d}}_{m}$ is the closest integer value to $d_{m}$, and ${\bar{d}}_{n}$ is defined in the same way.

For mathematical convenience, we only consider the case in the lifting structure of one-level 5/3 inverse MCTF in the following. Thus, according to the Equations (10) and (11), the errors in the reconstructed $f_{2i}(m,n)$ and $f_{2i+1}(m,n)$ can be formulated as:

(12) $$\varepsilon_{f_{2i}}=-\left(1/4\right)\cdot \varepsilon_{H_{i-1}}+1\cdot \varepsilon_{L_{i}}-\left(1/4\right)\varepsilon_{H_{i}},$$

(13) $$\varepsilon_{f_{2i+1}}=-\left(1/8\right)\cdot \varepsilon_{H_{i-1}}+\left(1/2\right)\cdot \varepsilon_{L_{i}}+\left(3/4\right)\cdot \varepsilon_{H_{i}}+\left(1/2\right)\cdot \varepsilon_{L_{i+1}}-\left(1/8\right)\cdot \varepsilon_{H_{i+1}},$$

where $\varepsilon_{f}$ denotes the reconstruction error of frame f. The assumption supposes that the distortion in each T-band can be modeled as independent and identically-distributed zero-mean additive white noise. Let $\sigma_{L}^{2}$ and $\sigma_{H}^{2}$ be the error variance of pixels in low-frequency subbands and that of pixels in high-frequency subbands, respectively. Also, let $\sigma_{f_{2i}}^{2}$ and $\sigma_{f_{2i+1}}^{2}$ denote the error variance in the reconstructed video frames. Accordingly, the distortions in the reconstructed frames are expressed as:

(14) $$\sigma_{f_{2i}}^{2}=1\cdot \sigma_{L}^{2}+(1/8)\cdot \sigma_{H}^{2},\;\sigma_{f_{2i+1}}^{2}=(1/2)\cdot \sigma_{L}^{2}+(19/32)\cdot \sigma_{H}^{2}.$$

The distortion associated with different T-bands will contribute differently to distortion in the reconstructed frames. Typically, this difference is quantified by analyzing the weighting factor for inverse MCTF. The weighting factor indicates how much a unit distortion in a specified subband contributes to the overall distortion in the reconstructed video. The derivation of its weighting factors is given as:

(15) $$\omega_{L}={\left(1/2\right)}^{2}+{\left(1\right)}^{2}+{\left(1/2\right)}^{2}=1.5,$$

(16) $$\omega_{H}={\left(-1/8\right)}^{2}+{\left(-1/4\right)}^{2}+{\left(3/4\right)}^{2}+{\left(-1/4\right)}^{2}+{\left(-1/8\right)}^{2}\approx 0.7188.$$

This results in distortion fluctuation across the reconstructed frames after one-level inverse MCTF. When multi-level MCTF is involved, the weighting factor of each temporal subband is modeled as:

(17) $$\omega_{LL}=\omega_{L}\cdot \omega_{L},\;\omega_{LH}=\omega_{L}\cdot \omega_{H},\;\omega_{LLL}=\omega_{LL}\cdot \omega_{L},\;\omega_{LLH}=\omega_{LL}\cdot \omega_{H},\ldots$$

Therefore, the distortion relationship between the T-bands and the reconstructed frame can be derived in the same way but with an iterative calculation. That is, the distortion for the original video sequences (denoted as D) after T levels MCTF decomposition can be derived by:

(18) $$\begin{array}{l} D=\sum_{i=1}^{K}D_{i}\\ \;=\sum_{i=1}^{K}\left(\sum_{t=1}^{T}\overset{t-1}{\underset{j=1}{\prod }}\omega_{i\_H}^{\left(j\right)}d_{i\_H}^{\left(t\right)}+\overset{T}{\underset{t=1}{\prod }}\omega_{i\_L}^{\left(t\right)}d_{i\_L}^{\left(T\right)}\right) \end{array},$$

where $d_{i\_H}^{\left(t\right)}$, and $d_{i\_L}^{\left(T\right)}$ are the reconstruction distortion (namely error variance) of the high-frequency subbands in the tth (1≤t≤T) temporal level, and low-frequency subbands in the highest temporal level, respectively. Additionally, $\omega_{i\_H}^{\left(j\right)}$ and $\omega_{i\_L}^{\left(t\right)}$ represent the weighting factors dependent on the motion estimation algorithm and the wavelet filter pair used for MCTF.

### 3.3. Adaptive Lagrange Multiplier Selection

As described in Subsection 2.2, the Lagrange multiplier λ is usually used to guide the bit allocation of each subband so that the overall distortion can be minimized and also with balanced the rate and distortion among the reconstructed frames. To our best knowledge, a larger λ will result in a higher coding distortion with less coding bits. Contrarily, a smaller λ will lead to a lower coding distortion with more coding bits. Therefore, we can allocate more bits to the T-bands with more detailed information by decreasing the subband-level Lagrange multiplier.

We define a weighting factor vector as $\Omega =\left(\omega_{1},\omega_{2},\ldots \omega_{K}\right)$, which indicates the contribution of a unit quantization error in each temporal subband to the overall distortion in the reconstructed video sequence. To obtain the weighting factor of the MCTF process, we need to employ a novel derivation, which involves the complicated motion compensation process and temporal subband content characteristics statistics. For computing these weighting factors, we utilize the mutual information, gradient per pixel, and texture homogeneity to measure the temporal subband content features.

Mutual information (MI) is a measurement of similarity between frames which can detect the differences among successive frames [44]. In this paper, we utilize MI to measure the similarity between the temporal subband frames. A large difference between frames (corresponding to the high motion activity) leads to a low MI value, while a small change between frames responds to a high MI value [45,46].

Let X be a discrete random variable with a set of possible outcomes $A_{X}=\left\{a_{1},a_{2},\ldots ,a_{L}\right\}$ with possibilities $\left\{p_{1},p_{2},\ldots ,p_{L}\right\}$, $p_{X}\left(x=a_{l}\right)=p_{ll}\geq 0$, and $\sum_{x\in A_{X}}p_{X}(x)=1$. According to the information theory, the entropy of X is:

(19) $$H(X)=-\sum_{x\in A_{X}}p_{X}(x)\log p_{X}(x).$$

The joint entropy of discrete random variables X and Y is:

(20) $$H(X,Y)=-\sum_{x,y\in A_{X},A_{Y}}p_{XY}(x,y)\log\left[p_{XY}\left(x,y\right)\right].$$

The MI between random variable X and Y is given by:

(21) $$I(X,Y)=-\sum_{x,y\in A_{x},A_{y}}p_{XY}(x,y)\log\frac{p_{XY}(x,y)}{p_{X}(x)p_{Y}(y)}.$$

The relation between MI and joint entropy is given by:

(22) I(X,Y)=H(X)+H(Y)−H(X,Y).

In a YUV formatted video sequence, let us consider a gray level video sequence with intensity value ranging from 0 to L−1 (e.g., L = 256 for 8-bit depth). For the luminance component Y, $P_{i,i+1}^{Y}(x,y)\;\left(0\leq x,y\leq L-1\right)$ is the probability that a pixel with gray level x in frame $f_{i}$ has a gray level y in frame $f_{i+1}$. Let $MI_{i,i+1}^{Y}$ be the MI of the luminance component. So we can obtain the $MI_{i,i+1}^{Y}$ value as shown below:

(23) $$MI_{i,i+1}^{Y}=-\sum_{x=0}^{L-1}\sum_{y=0}^{L-1}P_{i,i+1}^{Y}(x,y)\log\left[P_{i,i+1}^{Y}(x,y)/P_{i}^{Y}(x)P_{i+1}^{Y}(y)\right],$$

where $P_{i}^{Y}(x)$ and $P_{i+1}^{Y}(y)$ are the possibilities that a pixel with gray level x in frame $f_{i}$ and a pixel with gray level y in frame $f_{i+1}$, respectively.

The gradient based subband content complexity measure has been considered in our Lagrange multiplier selection scheme. Here, the gradient per pixel (GPP) of the temporal subband is defined by:

(24) $$GPP_{i}=\frac{1}{M\times N}\sum_{n=0}^{N-1}\sum_{m=0}^{M-1}\left(\left|I_{i}\left(m,n\right)-I_{i}\left(m+1,n\right)\right|\right)+\left(\left|I_{i}(m,n)-I_{i}(m,n+1)\right|\right),$$

where M and N are the width and height of the temporal subband, and $I_{i}(m,n)$ is the pixel value at position (m,n).

With the statistical analysis, we observe that, if the subband belongs to the homogeneous regions, the number of bits will be a smaller value, and it will relevantly increase with the complex of the texture. Hence, the texture homogeneity of each subband is measured by calculating the ratio between variance and mean of the subband, which is given by:

(25) $$Ratio_{k}=\frac{Variance}{Mean}=\frac{Variance}{\frac{1}{256}\sum_{n=0}^{N-1}\sum_{m=0}^{M-1}I_{i}\left(m,n\right)},$$

where Variance and Mean are the variance and mean value of each temporal subband, respectively.

Let W denote the synthesis gain matrix with regard to target video sequence. Therefore, the associated temporal subband weighting factor $\Omega =\left(\omega_{1},\omega_{2},\ldots \omega_{K}\right)$ for prediction can be calculated as:

(26) $$\omega_{i}=\omega_{filter\_i}\cdot {\left(\alpha \cdot GPP_{i}+\beta \cdot Ratio_{i}\right)}^{-\gamma \cdot MI_{i}}\cdot W_{i\to i+1},$$

where α, β, and γ are model parameters updated by multiple linear regression analysis. For each temporal subband frame i, $\omega_{filter\_i}$ is the filter-based weighting factor using Equations (15)–(17); $MI_{i}$, $GPP_{i}$, and $Ratio_{i}$ can be computed by Equations (23)–(25), respectively. As pointed out in [31], $W_{i\to i+1}$ is the cross-subband error propagation, which reflects the importance of coefficients in each subband.

Similar to JPEG2000 [47], we assume that the distortions from various temporal subbands are approximately additive. Equation (18) reveals that the total distortion is simply a linear combination of the distortions of all the temporal subbands. It is noticeable that the wavelet coefficients within each temporal subband obey the Gaussian distribution [48,49], hence, the distortion of the transform coefficients can be expressed as follows:

(27) $$d_{i}=k^{\prime }\frac{\pi e}{6}{\left(\prod_{q=1}^{Q}\sigma_{q}^{2}\right)}^{1/Q}2^{-\eta r_{i}}\omega_{i},$$

where $d_{i}$ and $r_{i}$ stand for the distortion and bitrate for the ith temporal subband frame, respectively. $\sigma_{q}^{2}$ is the variance of the qth coefficient, Q the number of coefficients concerned. Besides, $k^{\prime }$ and η are the parameters related to the temporal decomposition scheme used and the corresponding slope for SVC, respectively. 

However, in practice, it is very difficult to determine the variance for a given coefficient, as required by Equation (27). Based on the observation the variance of each coefficient can be estimated by:

(28) $$\sigma_{q}^{2}=\beta_{q}\sigma_{f}^{2},$$

where $\beta_{q}$ is a parameter related to the wavelet transform, and $\sigma_{f}^{2}$ the variance of the residual pixel values before wavelet transform. Therefore, Equation (27) can then be rewritten as:

(29) $$d_{i}=k^{\prime }\cdot \frac{\pi e}{6}\cdot {\left(\prod_{q=1}^{Q}\beta_{q}\sigma_{f}^{2}\right)}^{1/Q}\cdot 2^{-\eta r_{i}}\cdot \omega_{i}.$$

Moreover, the variance of the residual pixel value before wavelet transform $\sigma_{f}^{2}$ can be further approximated using the mean absolute difference (MAD) by $\sigma_{f}^{2}\approx 2MAD^{2}$. Consequently, the distortion $d_{i}$ for the i th subband can be expressed as:

(30) $$\begin{array}{ll} d_{i} & =k^{\prime }\cdot \frac{\pi e}{6}\cdot {\left(\prod_{q=1}^{Q}\beta_{q}\sigma_{f}^{2}\right)}^{1/Q}\cdot 2^{-\eta r_{i}}\cdot \omega_{i}\\ & =k^{\prime }\cdot \frac{\pi e}{6}\cdot {\left(\prod_{q=1}^{Q}\beta_{q}\cdot 2MAD^{2}\right)}^{1/Q}\cdot 2^{-\eta r_{i}}\cdot \omega_{i}\\ & =k\cdot \frac{\pi e}{6}\cdot MAD^{2}\cdot 2^{-\eta r_{i}}\cdot \omega_{i} \end{array},$$

with $k=2k^{\prime }{\left(\prod_{q=1}^{Q}\beta_{q}\right)}^{1/Q}$ a parameter related to the wavelet transform decomposition scheme used.

According to Equations (26)–(30), it is easy to compute the Lagrange multiplier $\lambda_{i}$ of the ith subband as:

(31) $$\lambda_{i}=-{\frac{\partial d_{i}}{\partial r_{i}}|}_{r_{i}=R_{i}},\;(i=1,2,\ldots K),$$

Correspondingly, Equation (31) can be written as:

(32) $$\{\begin{array}{c} \lambda_{1}=-\omega_{1}\cdot 2^{-\eta }\cdot {\frac{\partial d_{1}}{\partial r_{1}}|}_{r_{1}=R_{1}}\\ \lambda_{2}=-\omega_{2}\cdot 2^{-\eta }\cdot {\frac{\partial d_{2}}{\partial r_{2}}|}_{r_{2}=R_{2}}\\ \vdots \\ \lambda_{K}=-\omega_{K}\cdot 2^{-\eta }\cdot {\frac{\partial d_{K}}{\partial r_{K}}|}_{r_{K}=R_{K}} \end{array},$$

Based on Equations (30)–(32), a more practical Lagrange multiplier expression is then derived as:

(33) $$\lambda_{i}=\varphi MAD^{2}2^{-\eta r_{i}}\omega_{i},$$

with φ=6.2 an empirical constant suitable for different sequences.

By means of Lagrange multiplier $\lambda_{i}$, the RDO objective function Equation (7) can be rewritten as:

(34) $$\begin{array}{ll} J(F_{i}) & =D_{i}(F_{i},M_{i})+\lambda_{i}\cdot R_{i}(F_{i},M_{i})\\ & =\sum_{i=1}^{K}\omega_{i}d_{i}(r_{i})+\lambda_{i}(\sum_{i=1}^{K}R_{i}-R_{T})\\ & =\sum_{i=1}^{K}\left[\sum_{t=1}^{T}\overset{t-1}{\underset{j=1}{\prod }}\omega_{i\_H}^{\left(j\right)}d_{i\_H}^{\left(t\right)}(r_{i})+\overset{T}{\underset{t=1}{\prod }}\omega_{i\_L}^{\left(t\right)}d_{i\_L}^{\left(T\right)}(r_{i})\right]+\lambda_{i}\cdot (\sum_{i=1}^{K}R_{i}-R_{T}) \end{array},$$

where $\omega_{i\_H}^{\left(j\right)}d_{i\_H}^{\left(t\right)}(R_{i})$ denote the weighted distortion term for the high-frequency subbands, and $\omega_{i\_L}^{\left(t\right)}d_{i\_L}^{\left(T\right)}(R_{i})$ the weighted distortion term for the low-frequency subbands. Only the frame mode with the minimum Lagrange cost $J\left(F_{i}\right)$ is finally chosen as the best frame mode.

### 3.4. Summary of the Proposed Algorithm

The detailed procedure of the proposed Lagrange multiplier selection algorithm is outlined in the following Algorithm 1. 

**Algorithm 1 An efficient algorithm for content adaptive Lagrange multiplier selection****Step** **1**Initialization: Input a video sequence with K frames for illustration. Obtain the frame rate, total number of frames, target bitrate, etc., from the configuration file or manual input.**Step** **2**Modeling the distortion relationship between the temporal subbands and reconstructed frames.**Step** **3**Temporal subband weighting factor calculation (for temporal subband i)(3-1)Calculate MI value $MI_{i,i+1}^{Y}$ of luminance component between $f_{i}$ and $f_{i+1}$ by Equation (23);(3-2)$GPP_{k}$ calculation for each subband according to Equation (24);(3-3)Texture homogeneity $Ratio_{k}$ calculation for each subband according to Equation (25);(3-4)Apply $MI_{i,i+1}^{Y}$, $GPP_{k}$, and $Ratio_{k}$ into Equation (26) to compute temporal subband weighting factor $\omega_{i}$.**Step** **4**Lagrange multiplier selection: Carry out the adaptive Lagrange multiplier selection scheme by means of Equations (30)–(33).**Step** **5**Model parameters update: Parameters update using multiple linear regression analysis.**Step** **6**Temporal subband frame mode selection:(6-1)Calculate the Lagrange cost $J(F_{i})$ for the temporal subband frame i with mode $M=(m_{1},m_{2},m_{3},m_{4})$.(6-2)Output the best frame mode $M^{*}$ with the minimum cost $J(F_{i})$.**Step** **7**Loop until all frames are encoded:Set i:=i+1, if i<K, going to **Step 2** until the end of encoding, else report the related encoding parameters and exit.

In order to access the accuracy of the proposed R−λ model, $R^{2}$ is utilized as the quantitative metric which can measure the degree of data variation from a given model [50]:

(35) $$R^{2}=1-\frac{\sum_{i}{\left(X_{i}-{\hat{X}}_{i}\right)}^{2}}{\sum_{i}{\left(X_{i}-{\bar{X}}_{i}\right)}^{2}},$$

where $X_{i}$ and ${\hat{X}}_{i}$, respectively, are the actual and the estimated values of the ith data point. $\bar{X}$ is the mean of all the data points. The maximum $R^{2}$ value is 1, which occurs when $X_{i}={\hat{X}}_{i}$ for any i. The closer the value of $R^{2}$ is to 1, the more accurate the model is. In our experiment, for each test sequence, the λ value of each MCTF level is fitted at the target bitrate by the proposed R−λ model. The $R^{2}$ statistics for four-level MCTF are tabulated in Table 2. From this table, we can notice that the $R^{2}$ values are all close to 1. Consequently, it can be concluded that the analytical R−λ model works well for sequences at the different temporal decomposition levels. This is a crucial point of our approach.

## 4. Experimental Results

In this section, extensive experiments have been conducted to verify the effectiveness of the proposed content adaptive Lagrange multiplier selection algorithm for 3-D wavelet-based SVC.

### 4.1. Video Test Sequences

**Figure 3:**
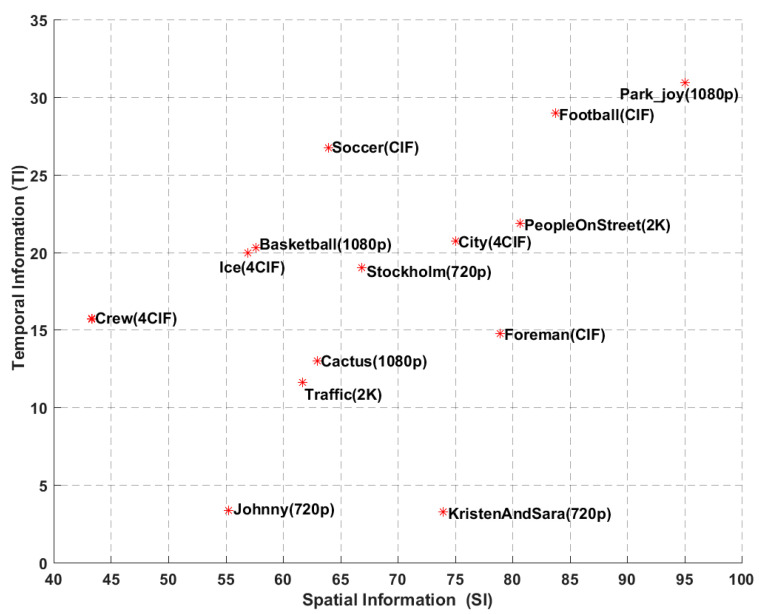
The spatial and temporal information indices of the test sequences (red star represents the coordinate value of SI and TI in the test sequence).

The experimental results over all video coding schemes are reported for fourteen standard test sequences based on YUV color format with 4:2:0 color sampling and 8 bits of precision per sample, including five different resolutions: CIF (352 × 288, 30 fps), 4CIF (704 × 576, 30 fps), 720p (1280 × 720, 60 fps), 1080p (1920 × 1080, 50 fps), and 2K (2560 × 1600, 30 fps), which are listed in Table 3. We also plot the temporal information (TI) and spatial information (SI) indices [51,52] of all source sequences in Figure 3. It demonstrates that the test sequences cover a wide range of video contents in terms of motion and spatial details. For each of the resolutions, the test sequences are decoded at different target bitrates (CIF and 4CIF: 256, 384, 512, 640, 768, 896, 1024 kbps; 720p: 384, 512, 640, 768, 896, 1024, 1536 kbps; 1080p and 2K: 2048, 3072, 4096, 5120, 6144, 7168, 10240 kbps).

### 4.2. Experimental Setup

In this paper, all the algorithms are implemented with ANSI C in Microsoft Visual C++ 6.0 and MATLAB R2012b programming environments. Our experiments are conducted on a 4-core (i5-2400@3.10GHz) computer equipped with RAM 8 GB that is also used to measure the computational complexity of our method. In the simulation, each group of pictures (GOP) contains 16 frames. During the MCTF process, motion estimation is implemented using a full-search with quarter–pixel accuracy on the dyadic wavelet coefficients. The block size varies from 4 × 4 to 128 × 128, and the search range of both the horizontal and vertical dimension is [–16, 15]. The default Lagrange multiplier values are given in the rate-distortion optimized mode selection process. All the other encoder settings are set identically for all methods.

### 4.3. Performance Evaluation

To evaluate the effectiveness of our algorithm, we have implemented it on the 3-D wavelet-based SVC reference software ENH-MC-EZBC configured with the common test conditions as suggested in configure file. In the simulation, we have performed the following five representative codecs with the same configuration. Recently, these codecs deliver the best coding performance for scalable coding of video datasets [53]:

- MC-EZBC: the original MC-EZBC without employing proper RDO scheme.
- RPI-MC-EZBC: the bidirectional MC-EZBC from Rensselaer Polytechnic Institute which uses Haar filters for the conventional MCTF framework with the default Lagrange multiplier value for all the temporal decomposition levels.
- RWTH-MC-EZBC: the improved version of MC-EZBC from RWTH Aachen University, which uses longer filters instead of Haar filters for the conventional MCTF framework with the corresponding fixed Lagrange multiplier for each temporal decomposition level.
- ENH-MC-EZBC: the enhanced MC-EZBC using an adaptive MCTF framework with the corresponding fixed Lagrange multiplier for each temporal decomposition level.
- Proposed method: our codec with the proposed content adaptive Lagrange multiplier selection method.

In the experiments, only the luminance component is taken into consideration since human visual system is less sensitive to color than to luminance. For reasons of brevity, the average peak signal-to-noise ratios (PSNR) (dB) and the standard deviation of PSNR (PSNR STD) on luminance component have been used as quality metric, which are defined as follows:

(36) $$PSNR=10log_{10}\left(\frac{255^{2}}{MSE}\right),$$

(37) $$PSNR\;STD=\sqrt{\frac{K\cdot \sum_{i=0}^{K-1}PSNR_{i}^{2}-{\left(\sum_{i=0}^{K-1}PSNR_{i}\right)}^{2}}{K\cdot \left(K-1\right)}},$$

where MSE denotes the mean square error between the original frame and reconstructed frame, $PSNR_{i}$ stands for the PSNR of the ith reconstructed frame and K the number of video frames in a sequence. Note that a higher PSNR means that a better RD performance is achieved. Meanwhile, the smaller the PSNR STD value, the better the video quality perceived by the end user, and vice versa.

#### 4.3.1. Comparison of Rate-Distortion Performance

To verify the overall rate-distortion (R-D) performance of the proposed Lagrange multiplier selection approach, we compare it with the successful coding schemes on the framework of motion-compensated subband coding (MCSBC): ENH-MC-EZBC, RWTH-MC-EZBC, RPI-MC-EZBC, and MC-EZBC. Let “Scheme 1”, “Scheme 2”, “Scheme 3”and “Scheme 4” denote our method compared to the ENH-MC-EZBC, RWTH-MC-EZBC, RPI-MC-EZBC, and MC-EZBC, respectively.

**Figure 4:**
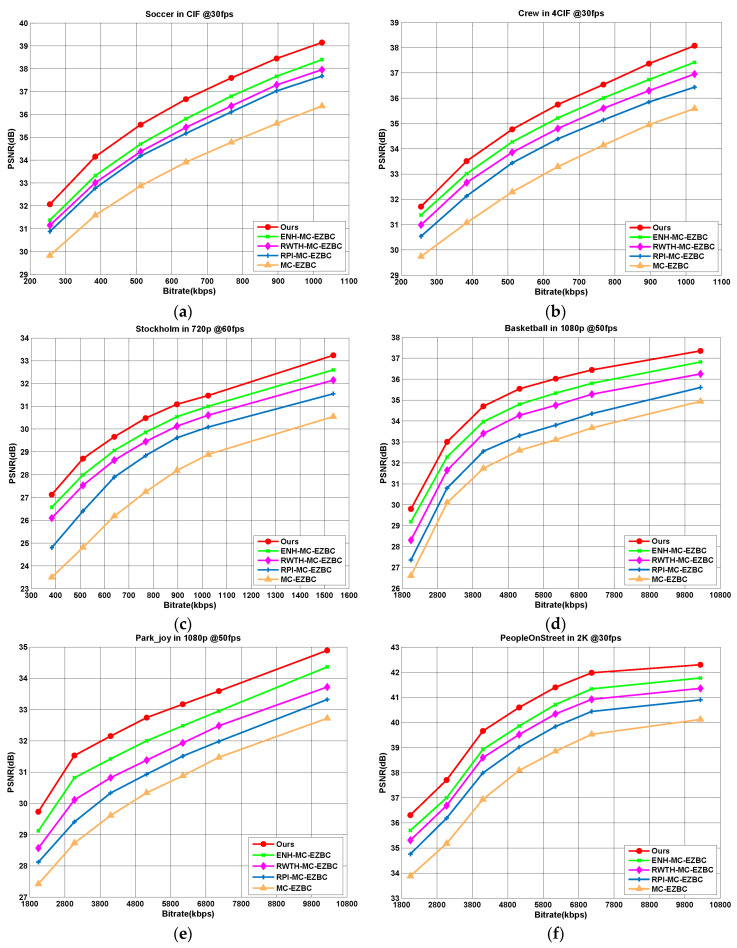
R-D performance comparisons among five different codecs for sequences: (**a**) Soccer; (**b**) Crew; (**c**) Stockholm; (**d**) Basketball; (**e**) Park_joy, and (**f**) PeopleOnStreet.

Figure 4 shows the R-D curves of five codecs for six selected test sequences (“Soccer”, “Crew”, “Stockholm”, “Basketball”, “Park_joy”, and “PeopleOnStreet”) at different target bitrates. Obviously, it is observed that the codec with the proposed algorithm achieves the best R-D performance among all codecs. From these figures, we can see that our method is noted with average PSNR improvement over other methods about 0.53–3.2 dB. As shown in Figure 4a, for the “Soccer” sequence, the proposed algorithm yields 0.79, 1.15, 1.4, and 2.7 dB higher PSNR values than the ENH-MC-EZBC, RWTH-MC-EZBC, RPI-MC-EZBC, and MC-EZBC in average, respectively.

**Figure 5:**
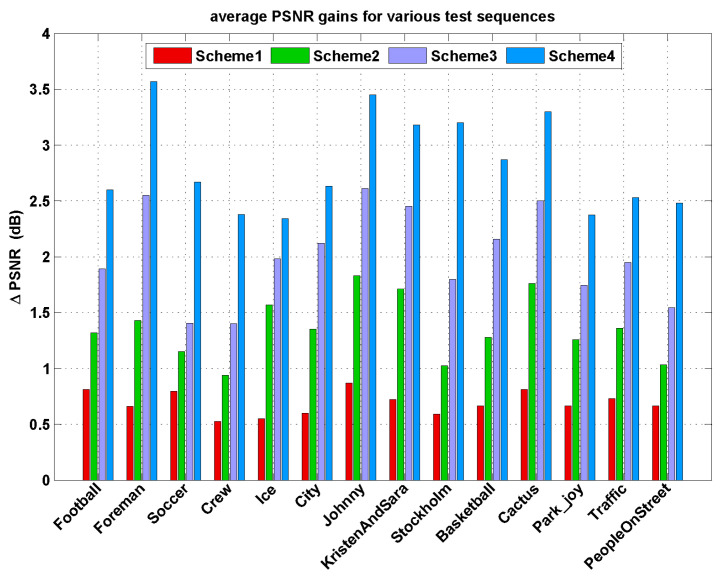
The average PSNR gains obtained for various test sequences.

Average PSNR gains for all the test sequences are also displayed in Figure 5, one can see that the proposed Lagrange multiplier algorithm achieves an average of 0.5–3.57 dB improvement in PSNR results. The PSNR gains are more especially significant for the test sequences with complex texture and/or highly moving objects, such as “Foreman”, “Soccer”, “City”, “Stockholm”, “Basketball”, and “Park_joy” sequences. These test sequences contain abrupt changes over frames in video content characteristics with fast moving objects and highly spatial details and tend to be encoded with various coding types. Thus, for all target bitrates ranges, our method shows the remarkable superiorities of RD performances with higher PSNR gains by performing content-adaptive Lagrange multiplier selection.

**Figure 6:**
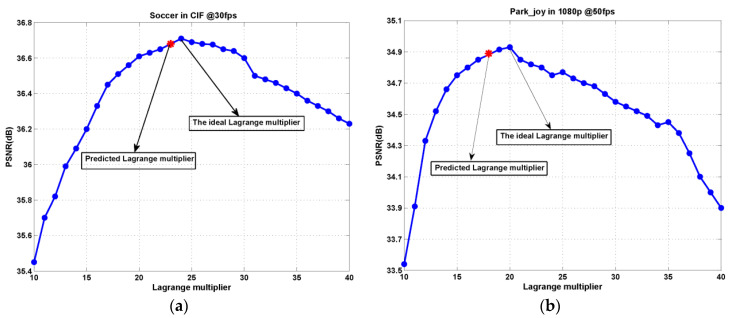
Average PSNR versus Lagrange multiplier at different target bitrates for test video sequences: (**a**) Soccer (640 kbps) and (**b**) Park_joy (10240 kbps).

Figure 6 shows the average PSNR of test video sequences “Soccer” and “Park_joy” at different target bitrates for various Lagrange multiplier values, where the ideal points and the points obtained by the proposed Lagrange multiplier selection are respectively marked. The observation indicates again that the proposed Lagrange multiplier results in the near-ideal rate-distortion performance for 3-D wavelet-based SVC at different target bitrates.

**Figure 7:**
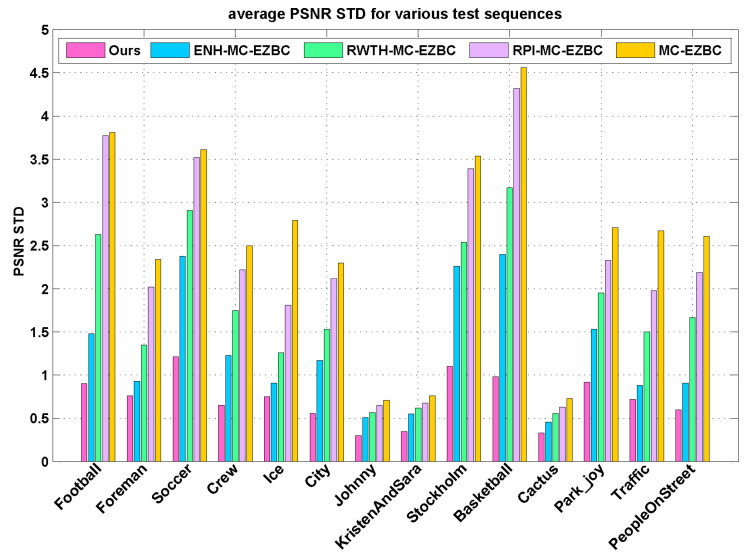
The average standard deviations of PSNR for various test sequences.

In addition, we also investigate PSNR variations during video reconstruction. Since high fluctuation of frame PSNR values may cause perceptual annoying to viewers, the fluctuation of PSNR values is one of the vital factors for video coding applications. The standard deviation of PSNR (PSNR STD) is utilized to measure for the smoothness of video quality. The smaller PSNR STD value result in the smoother PSNR variation and hence more consistent video quality over the video frames. Figure 7 illustrates the average PSNR STD values over different target bitrates for the proposed algorithm and the other four methods. Compared to the four methods, the proposed algorithm achieves more stable visual quality with substantially smaller PSNR fluctuations as depicted in Figure 7. From this figure, we can see that the proposed Lagrange multiplier selection algorithm reduces the PSNR standard deviations of all frames by up to 3.58 with respect to RPI-MC-EZBC. As shown in Figure 7, our method generates the minimum PSNR fluctuation over the entire sequence than the other three methods. Hence, we reach the conclusion that the proposed method can be beneficial for controlling the fluctuations of qualities in the reconstructed video and produce more stable visual quality than others.

#### 4.3.2. Comparison of Subjective Performance

**Figure 8:**
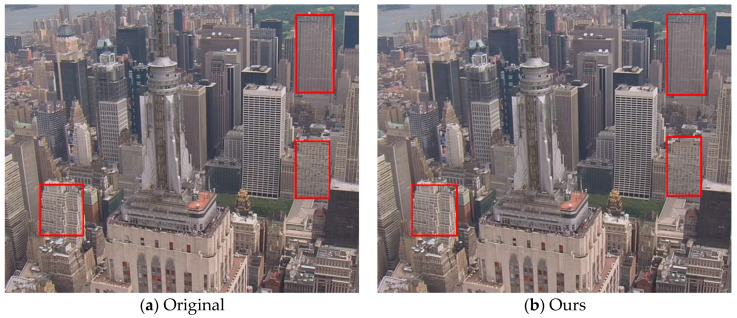

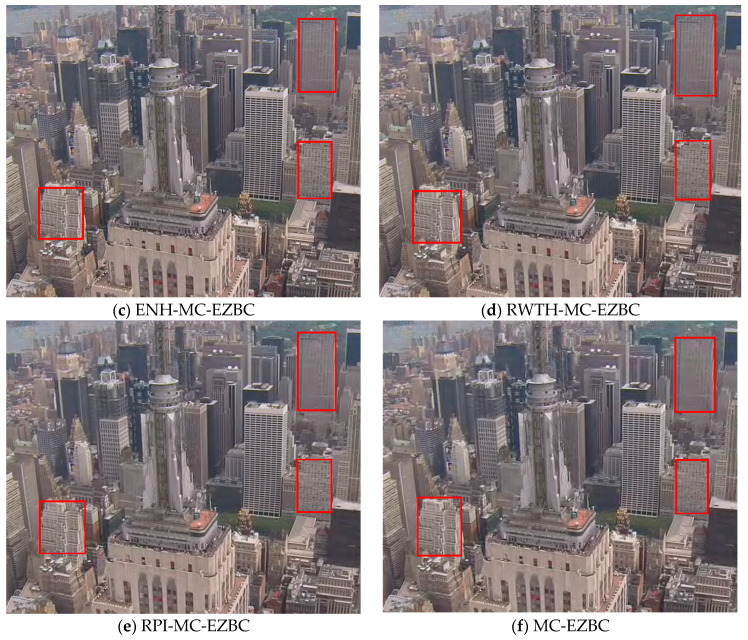
Subjective visual quality comparisons of the 8th reconstructed frame of “City” sequence at 896 kbps.

To obtain the subjective inspection of the reconstructed frames, we show the 8th reconstructed frame of the “City” sequence at 896 kbps in Figure 8. From this figure, we can see that the visual quality of the reconstructed frame by the proposed method is conspicuously better than those by the other four methods. It can be plainly discerned that the frame processed by our codec presents less blocking artifacts in the homogeneous regions, better preserved textures, and sharper appearance than other codecs. In particular, it is worth noting that the regions with high spatial details (those enclosed by red rectangles on buildings) are well preserved by the proposed algorithm whereas they are not very clear by other reference codecs.

#### 4.3.3. Comparison of Computational Complexity

To measure the computational complexity, we define the encoding speed as the number of frames which can be encoded in one second on the hardware platform with processor Intel Core i5 4-core CPU 3.10GHz. The computational complexity is considered as inverse value of the encoding speed, which is measured without any use of assemblers, threads, or other program optimization techniques [6,7,15,28]. Table 4 demonstrates the encoding speed for the proposed algorithm, ENH-MC-EZBC, RWTH-MC-EZBC, RPI-MC-EZBC, and MC-EZBC. As shown in Table 4, the encoding speed of the proposed algorithm is about 1.14, 3.65, 4.65 times faster than RWTH-MC-EZBC, RPI-MC-EZBC, and MC-EZBC, respectively. The reason is that for the sequences with strong inter-frame dependencies, more bits are allocated to the reference frames which lead to better prediction results. Thus, only small residual signals need to be processed in the following encoding steps, which further result in computational complexity reductions. However, the encoding speed of ENH-MC-EZBC is about 1.11 times faster than our algorithm. This is mainly due to the additional operations on the statistics for video content characteristics. In general, the experimental results demonstrate that the computing overhead brought by the proposed Lagrange multiplier selection algorithm can be negligible when compared to the other four methods. Although our algorithm encodes at average 3.07 frames per second, it is insufficient for real-time video processing applications. 

Since the ENH-MC-EZBC reference software is non-optimized C++ implementation, development of an efficient low-complexity 3-D wavelet-based scalable video coding scheme is an important practical problem, which is necessary to be considered in our future work.

## 5. Conclusions

In this paper, we present an efficient content adaptive Lagrange multiplier selection algorithm for RDO in 3-D wavelet-based SVC. The wavelet filter types, subband coupling in the MCTF process, and temporal subband content characteristics have been incorporated into our algorithm to select the Lagrange multiplier adaptively. The simulation results demonstrate that the proposed algorithm turns out to be much better than the reference methods in terms of both accuracy and effectiveness.

In a future work, we are going to extend our algorithm to other scalabilities, not merely quality scalability. The overall video quality can be further improved by employing human visual system-based perceptual features. In addition, development of a scalable low-complexity video codec based on 3-D DWT is our main concern as well. Therefore, we will experiment in these directions to obtain a more compelling result.

## Figures and Tables

**Table 1:** Coefficients $\alpha_{\tau }$ and $\beta_{\tau }$ depending on the mode of the current frame.

Frame Mode	$\alpha_{1}$	$\alpha_{2}$	$\alpha_{3}$	$\beta_{1}$	$\beta_{2}$	$\beta_{3}$
bi-direction	−1/2	1	−1/2	1/4	1	1/4
uni-left	−1	1	0	1/2	1	0
uni-right	0	1	−1	0	1	1/2
intra	N/A	N/A	N/A	N/A	1	N/A

**Table 2:** The $R^{2}$ values of each MCTF level for test video sequences.

Sequences	MCTF Level			
	1st	2nd	3rd	4th
Football	0.9921	0.9894	0.9951	0.9975
Foreman	0.9860	0.9954	0.9967	0.9982
Soccer	0.9984	0.9962	0.9981	0.9993
Crew	0.9976	0.9983	0.9989	0.9991
Ice	0.9787	0.9899	0.9935	0.9980
City	0.9843	0.9894	0.9932	0.9982
Johnny	0.9885	0.9891	0.9964	0.9989
KristenAndSara	0.9932	0.9967	0.9970	0.9985
Stockholm	0.9924	0.9962	0.9984	0.9990
Basketball	0.9787	0.9812	0.9899	0.9988
Cactus	0.9949	0.9950	0.9966	0.9990
Park_joy	0.9815	0.9843	0.9957	0.9981
Traffic	0.9870	0.9932	0.9960	0.9991
PeopleOnStreet	0.9893	0.9919	0.9949	0.9993
**Average**	**0.9888**	**0.9919**	**0.9957**	**0.9986**

**Table 3:** Properties of standard video test sequences.

Sequences	Resolution	$K^{1}$	Characteristics
Football	352 × 288	260	Fast camera and human subject motion, highly spatial details
Foreman	352 × 288	300	Fast camera and content motion with pan at the end
Soccer	352 × 288	300	Fast changes in motion, rapid camera panning
Crew	704 × 576	300	Multiple moderate objects movement
Ice	704 × 576	240	Still background and moderate human subject motion
City	704 × 576	300	Fast camera motion, high detail of buildings
Johnny	1280 × 720	100	Still background and low local motion
KristenAndSara	1280 × 720	100	Still background and moderate local motion
Stockholm	1280 × 720	100	Moderate camera panning, high detail of buildings
Basketball	1920 × 1080	100	Fast camera and human subject motion, highly spatial details
Cactus	1920 × 1080	100	Circling motion and highly spatial details
Park_joy	1920 × 1080	100	Camera and content motion, high detail of trees
Traffic	2560 × 1600	100	Moderate translational motion and highly spatial details
PeopleOnStreet	2560 × 1600	100	Still background and many human subject motion

^1^ The number of frames in the test video sequence.

**Table 4:** Encoding speed comparison results.

Resolution	Encoding Speed				
	Ours	ENH-MC-EZBC	RWTH-MC-EZBC	RPI-MC-EZBC	MC-EZBC
CIF	5.31	5.82	4.87	1.52	1.21
4CIF	5.16	5.55	4.52	1.29	1.05
720p	3.05	3.57	2.71	0.87	0.64
1080p	1.09	1.34	0.83	0.35	0.29
2K	0.72	0.79	0.55	0.16	0.12
**Average**	**3.07**	**3.41**	**2.70**	**0.84**	**0.66**
